# An Alternative Technique for Fabrication of Frameworks in an Immediate Loading Implant Fixed Mandibular Prosthesis

**DOI:** 10.1155/2015/102189

**Published:** 2015-01-05

**Authors:** André Gustavo Paleari, Cristina Dupim Presoto, Juliano Alencar Vasconcelos, José Maurício dos Santos Nunes Reis, Lígia Antunes Pereira Pinelli, Regina Helena Barbosa Tavares da Silva, Cristiane Campos Costa Quishida

**Affiliations:** ^1^Department of Dental Materials and Prosthodontics, Araraquara Dental School, Universidade Estadual Paulista (UNESP), Rua Humaitá 1680, 14801-903 Araraquara, SP, Brazil; ^2^Department of Restorative Dentistry, Araraquara Dental School, Universidade Estadual Paulista (UNESP), Rua Humaitá 1680, 14801-903 Araraquara, SP, Brazil

## Abstract

The oral rehabilitation of edentulous patients with immediate loading has become a safe procedure with high predictability. The success is related to immediate fabrication of a passive fit framework to attach the implants. Based on these considerations, this case report shows an alternative technique for mandibular rehabilitation using implants immediately loaded, where the framework was fabricated using cylinders with internal reinforcement and precast pieces, electrowelding, and conventional welding providing esthetics and function to the patient in a short period of time.

## 1. Introduction

The oral rehabilitation of edentulous patients using implant-supported fixed prosthesis immediately loaded is a safe procedure with high predictability [1–5]. Such treatment has the advantages of immediate restoration of function and aesthetics and emotional comfort to patients, especially in cases of dental extractions and immediate implant placement. Moreover, few clinical sessions are required in view of the absence of the second surgical intervention for exposing the implants [1, 3, 6].

According to Romanos et al. [7], loading seems to initiate bone remodeling and to form new bone around immediately loaded implants, with a better healing of the hard and soft tissues. In addition, the implant-supported fixed complete prostheses are favored by biomechanical aspects of arrangement and polyhedral rigid framework connection uniting the implants, which improve the distribution of occlusal loads [8].

The rigid infrastructure prevents micromotion of the implants [9] and promotes primary stability and appropriate distribution of occlusal forces, since these are transmitted to the implants immediately after installation of the prosthesis [10]. Additionally, the fabrication of frameworks with passive adaptation to implant abutments promotes the maintenance of bone-implant interface [11–13]. The absence of passivity can cause serious complications such as bone loss and fracture of the abutments and screws [11, 12, 14].

Other factors that influence the success of rehabilitation with immediate loading are related to problems in casting that may occur as a result of the negligence of the dental technician during the steps involved in casting, as well as lack of knowledge about the materials and equipment used. Modifications or negligence in the process of casting or welding can cause alterations in the mechanical and structural properties of any alloy that is used for fabrication of the frameworks [14]. Furthermore, inaccuracies in the impression procedure and incorporation of air bubbles in the impression material can interfere with the precision of the master cast, generating a misfit framework [15–17].

The aim of this study is to report a case of an implant-supported mandibular prosthesis using a fast and efficient technique for the fabrication of the framework using cylinders with internal reinforcement and precast pieces, electrowelding, and conventional welding.

## 2. Case Report

**Figure 1:**
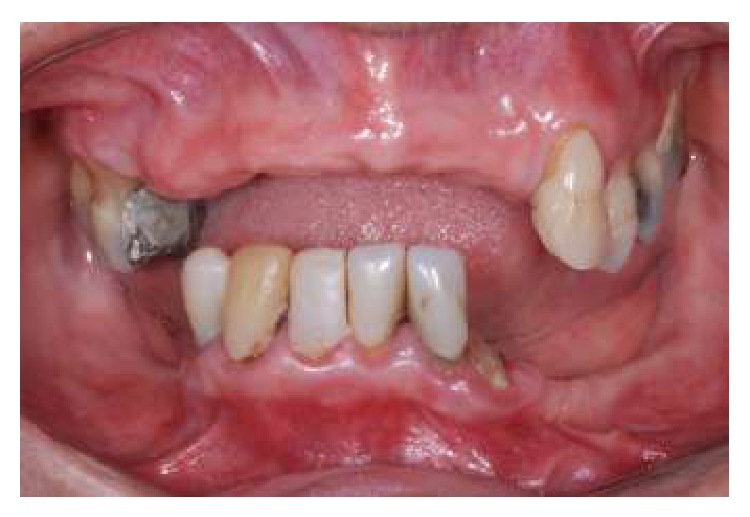
Initial clinical features.

The following clinical case presentation demonstrates the treatment of a 64-year-old female patient, who attended the Fixed Partial Denture Clinic of the Araraquara Dental School-Univ. Estadual Paulista (UNESP), for dental treatment. The patient presented clinically with tooth mobility and bone resorption in anterior mandibular arch. In addition, she was wearing an old and misfit removable partial dentures in the upper and lower arch and did not show any systemic disease. Given the clinical (Figure 1) and radiographic features, it was proposed to extract the remaining lower teeth and place an immediate loading implant-fixed mandibular prosthesis. This decision was made considering the bad conditions of mandibular teeth, which did not allow the rehabilitation with new removable partial dentures or another modality of dental prostheses. For the upper arch was proposed a provisional removable partial denture until the placement of bone graft with the purpose of subsequent placing dental implants.

**Figure 2:**
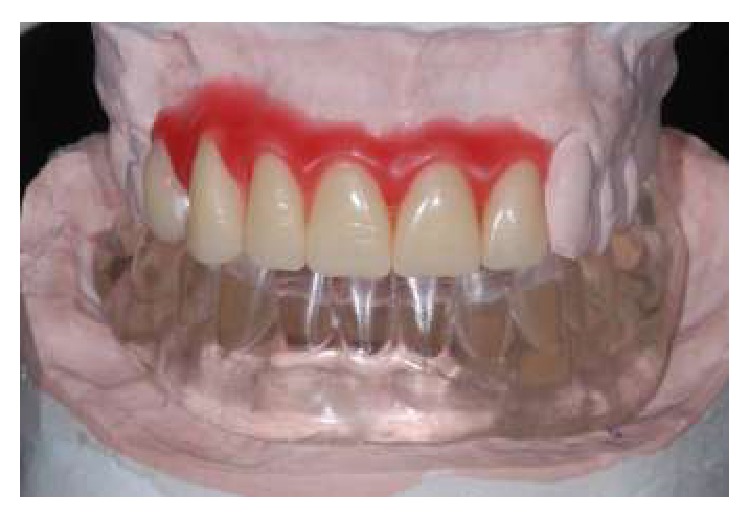
Multifunctional guide.

**Figure 3:**
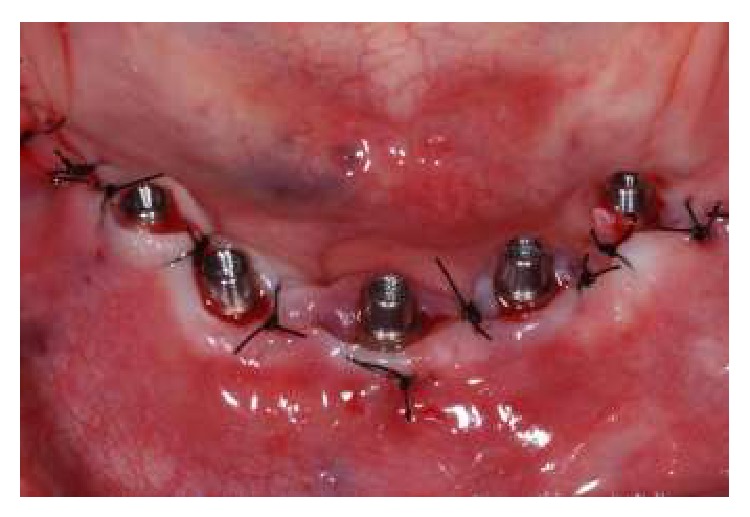
Abutments installed on the implants.

**Figure 4:**
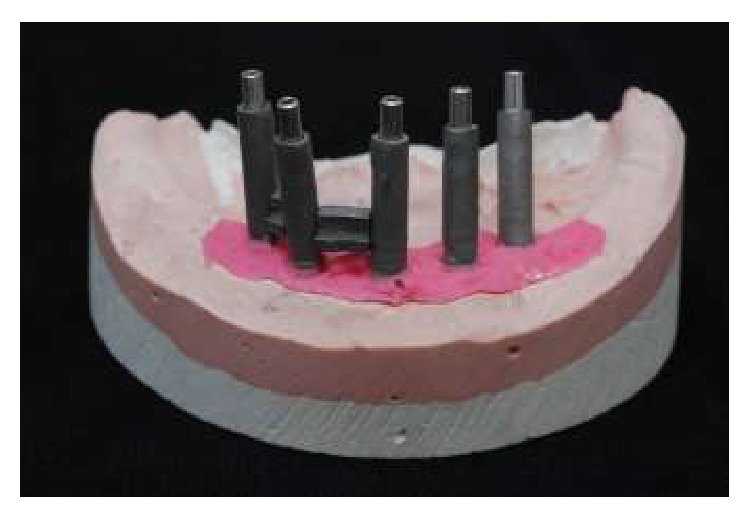
Fragments previously cast in Ni-Cr alloy inserted into the space between the cylinders.

In order to follow a protocol based on reverse planning, the intermaxillary relations were established and the casts mounted in semiadjustable articulator prior to implant placement. Thus, a multifunctional guide (Figure 2) was fabricated from a duplicate of the wax base, after the first clinical proof, to maintain the esthetic and dimensional features obtained previously. After the placement of the implants (Emfils-Indústria e Comércio de Produtos Odontológicos, Itu, SP, Brazil) with a greater torque than 40 N·cm, the abutments were installed (Figure 3) and an impression was performed using the multifunctional guide. Cylinders that were previously cast in Ni-Cr alloy (Fit Cast SB-Plus Ni-Cr without Beryllium, Talladium, Curitiba-PR, Brazil) were installed on the abutment replicas in the master cast. Fragments were earlier cast in Ni-Cr alloy and obtained by means of wax patterns and inserted into the space between the cylinders (Figure 4). The multifunctional guide was used as parameter to delimit the covering area to include the implants and set limits to the extent of the two distal cantilever extensions. The initial fixation of the metal fragments to cylinders was performed with an Electroweld (Kernit Mechatronics Ind. Ltd., Indaiatuba, Sao Paulo, Brazil) using orthodontic wire of 0.9 mm diameter. After primary stabilization, a conventional welding in Ni-Cr alloy was carried out, without the need to invest any wax pattern.

**Figure 5:**
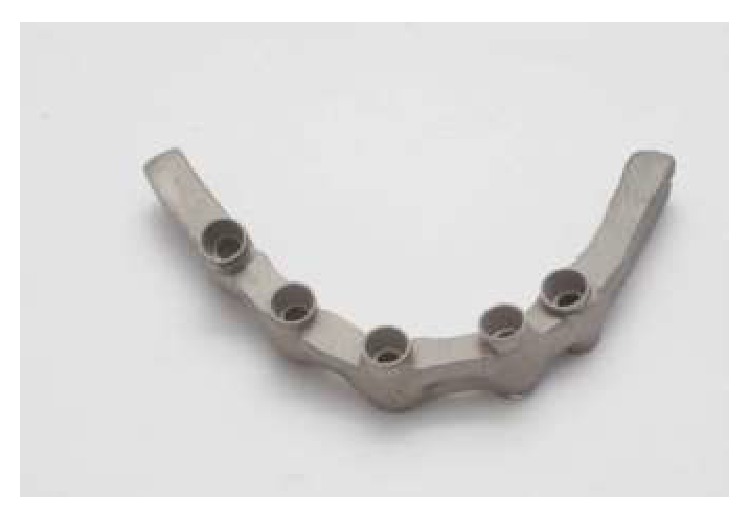
Framework after finishing.

**Figure 6:**
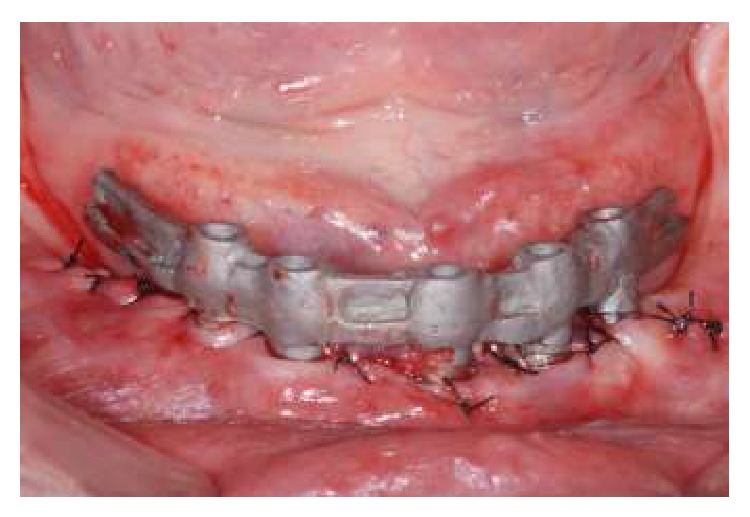
Framework try-in.

**Figure 7:**
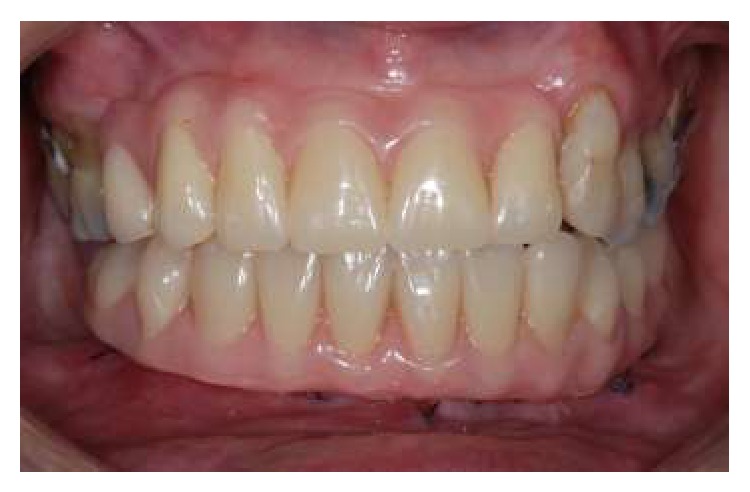
Prosthesis immediately after installation.

**Figure 8:**
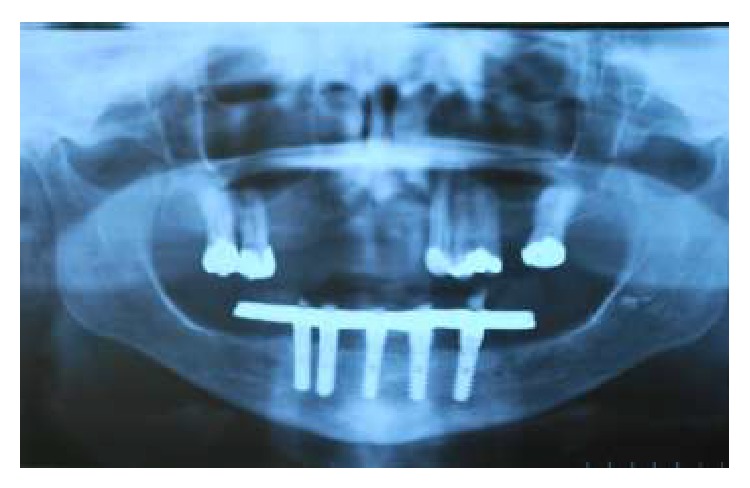
Panoramic radiograph after 12 months.

**Figure 9:**
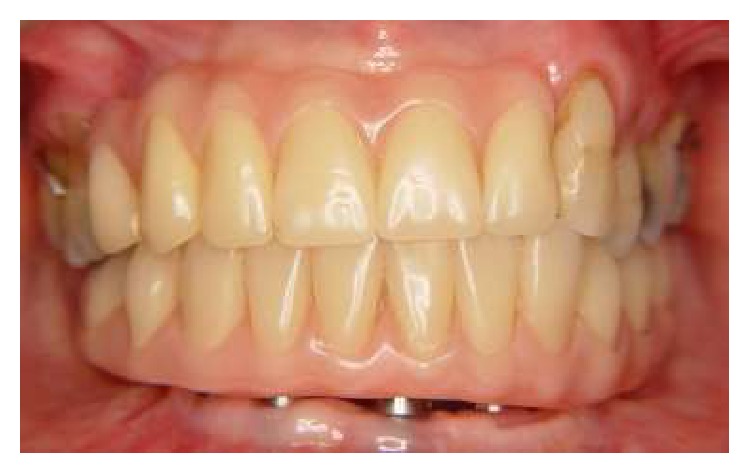
Clinical aspect of the prosthesis after 12 months.

Finally, the finishing in the framework with stones and disks and blasting with aluminum oxide (100 *μ*m) was performed (Figure 5). Then, it was performed the framework try-in on the abutments by first tightening down one of the terminal screws completely (Figure 6). After clinical and radiographic verification, the screw was unscrewed and the procedure was repeated for the other terminal abutment [18]. After these procedures, we performed a clinical evaluation of the teeth and, after the confirmation and approval of the patient, the prosthesis was installed in 12 hours after surgery (Figure 7). Figures 8 and 9 show the radiographic and clinical features of the prosthesis 12 months after the installation.

## 3. Discussion

The oral prosthetic rehabilitation using immediate loading implants has been reported as a beneficial treatment protocol to osseointegration of the implant and that increases the comfort of the patient. The surgical and prosthetic protocols have been developed in order to reduce the time between surgery and the installation of the prosthesis [5]. In addition, this treatment protocol has showed success rates similar to the treatments performed conventionally [3–5].

This case report presents a technique option to the fast confection of metal framework for the treatment of total edentulous patients when planning the implant-supported prostheses with immediate loading. In this technique, the metallic framework is fabricated using cylinders and fragments previously cast in Ni-Cr alloy, electrowelding, and conventional welding to promote reduction in manufacture time of the prosthesis, allowing it to be installed in the same day or within 24 hours after the surgery of the implant placement. The speed of the treatment is important mainly for patients who initially present natural teeth and are subjected to multiple extractions and procedures for implant placement with immediate loading [1]. Additionally, it has been reported that with immediately loaded implants the patients may restart their function quickly. Any reduction in the number of the surgical procedures necessary or a decrease in the healing period is certainly very well welcomed by clinicians and patients [19].

In this present case, the patient was not subjected to a second surgical phase or used a conventional complete mandibular denture during the period of osseointegration period. Based on clinical and tomographic features, it was decided to fabricate an implant-supported prosthesis with immediate loading, using the technique with cylinders and fragments of precast framework, electrowelding, and conventional welding. In this procedure, it is not necessary to wax and embedding the framework. Consequently, it can help to achieve a passive framework, which offers lower transmission of loads to the implants and peri-implant interface, thereby reducing the presence of harmful forces that can lead to bone loss around the implant, since passively fitting frameworks are a prerequisite for long-lasting osseointegration of dental implants [20]. The adaptation and passivity of the framework were verified by means of clinical probing and dental floss and confirmed by periapical radiography. However there is agreement among the in vitro studies that there is no implant framework fabrication approach or material that can provide absolute passivity of fitted frameworks [5, 11, 13, 21].

A panoramic radiograph performed 12 months after the installation of the prosthesis demonstrated satisfactory adaptation of the framework and bone integrity around the implants. This technique represents an alternative to the conventional procedure to treatment and rehabilitation of edentulous mandibles, providing satisfactory function and esthetics of the patient in a short period of time. However laboratory studies are needed to evaluate the adaptability and mechanical strength of frameworks fabricated by using this technique compared to conventional techniques. Furthermore, prospective controlled clinical trials should be designed to assess long term success of implant-fixed prosthesis fabricated by using this technique.
